# Exploration of Rice Husk Compost as an Alternate Organic Manure to Enhance the Productivity of Blackgram in *Typic Haplustalf* and *Typic Rhodustalf*

**DOI:** 10.3390/ijerph15020358

**Published:** 2018-02-17

**Authors:** Subramanium Thiyageshwari, Pandurangan Gayathri, Ramasamy Krishnamoorthy, Rangasamy Anandham, Diby Paul

**Affiliations:** 1 Department of Soils & Environment, Agricultural College and Research Institute, Tamil Nadu Agricultural University, Madurai 625 104, India; mani.gayathri3@gmail.com; 2 Department of Agricultural Microbiology, Agricultural College and Research Institute, Tamil Nadu Agricultural University, Madurai 625 104, India; moorthy.micro@gmail.com (R.K.); anandham@tnau.ac.in (R.A.); 3 Department of Environmental Engineering, Konkuk University, Gwanjin-Gu 143-701, Korea

**Keywords:** blackgram, cellulolytic microorganisms, compost, rice husk, microbial consortium

## Abstract

The present study was aimed at using cellulolytic bacterium *Enhydrobacter* and fungi *Aspergillus* sp. for preparing compost from rice husk (RH). Further, the prepared compost was tested for their effect on blackgram growth promotion along with different levels of recommended dose of fertilizer (RDF) in black soil (typic Haplustalf) and red soil (typic Rhodustalf) soil. The results revealed that, inoculation with lignocellulolytic fungus (LCF) *Aspergillus* sp. @ 2% was considered as the most efficient method of composting within a short period. Characterization of composted rice husk (CRH) was examined through scanning electron microscope (SEM) for identifying significant structural changes. At the end of composting, N, P and K content increased with decrease in CO_2_ evolution, C:N and C:P ratios. In comparison to inorganic fertilization, an increase in grain yield of 16% in typic Haplustalf and 17% in typic Rhodustalf soil over 100% RDF was obtained from the integrated application of CRH@ 5 t ha^−1^ with 50% RDF and biofertilizers. The crude protein content was maximum with the combined application of CRH, 50% RDF and biofertilizers of 20% and 21% in typic Haplustalf and typic Rhodustalf soils, respectively. Nutrient rich CRH has proved its efficiency on crop growth and soil fertility.

## 1. Introduction

In recent years, a huge amount of lignocellulosic waste is increasingly realized as an environmental problem and the utilization of these wastes becoming a welcome issue. Lignocellulosic waste is the most abundant natural waste substance and has become one of the most important raw materials for compost preparation. There has been much research focused on isolation of microorganisms which produces cellulase enzymes with maximum specific activities and efficiency [1]. However, there are only a few studies that focus on the utilization of these cellulolytic enzyme producing bacteria on compost preparation and subsequent usage [2].

The crop residues are abundant with cellulose, hemicellulose, lignin, pectin and also contain a low amount of diverse groups of substances like protein and fatty acids. The major portions of crop residues were burnt in the field itself. This results on one hand in a waste of renewable organic source in soil affecting C:N ratio and biota; on the other hand, burring of waste leads to the emission of greenhouse gases [3,4]. Rice husk is a by-product of the rice milling industry and it is an agricultural residue abundantly available in rice producing countries.

Bio-degradation of agricultural waste into compost and incorporation into soil may enhance the nutrient recycling and maintain soil fertility [5]. However, the presence of lignocellulosic content in the waste materials may slow down the microbial degradation process. Rice husk is generally not recommended as cattle feed since its cellulose and other sugar contents are low, which means there is a limited source of energy for the body. Moreover, rice husk (RH) contains a high C:N ratio of about 85:1 and is rich in silica and lignin which makes it difficult to be degraded. Utilization of raw RH or composed material is not utilized properly in agriculture for crop growth [6]. Keeping in mind the harmful effects of open field burning of rice husk, as well as the convenience of farmers, an economical, environment friendly and low labour intensive strategy should be adopted for effective utilization of rice husk. However, the presence of high lignin content makes the rice husk less vulnerable to microbial attack. For effective utilization of lingo–cellulosic residues, several physical and chemical pre-treatments are required, which may not be convenient for farmers of small holdings. To make the process of lignin and cellulose degradation economically viable, inoculation with lignocellulolytic microorganisms may prove beneficial [7].

Many lignocellulolytic bacteria were used for compost making from agricultural waste [8,2]. Fungus is widely used for composting of lignocellulosic waste because they are filamentous and have the ability to produce prolific spores that can invade substrates quickly. Fungal culture was used for the composting of rice straw which is one of the waste products from rice field [9]. The recycled material when applied to soil, improves soil fertility and health. The compost serves as an excellent source of nutrient in organic farming and mitigates the ill effects due to usage of chemical fertilizers.

Pulses are the cheapest source of dietary proteins. Blackgram is the third most important pulse crop in India; it has been distributed mainly in tropical to sub-tropical countries where it is grown mainly in the summer season. In India, blackgram is mainly grown in non-calcareous black soil of Typic Haplustalf and non-calcareous red soil of Typic Rhodustalf. Growth and yield attributes change among the soil types. Biofertilizers are cost effective, eco-friendly and a renewable source of plant nutrients in the sustainable agriculture system and are considered as a kingpin of modern agriculture. This undoubtedly boosted not only the food production but it also shows the positive effects on physicochemical properties of soil, nitrogen (N) transformation, macro and micro nutrients uptake and nutritional composition [10]. Hence, the present study was formulated to test the lignocellulose degrading microbial consortium for effective degradation of rice husk. Furthermore, compost obtained through effective microbial consortium was tested on blackgram growth promotion under pot culture condition in typic Haplustalf and typic Rhodustalf.

## 2. Materials and Methods

### 2.1. Composting Experiment

#### 2.1.1. Microorganisms, Earth Worm and Source of Plant Materials

The raw rice husk (RRH) was obtained from local rice mill, Madurai, Tamil Nadu, India. The ligncellulolytic bacterium (*Enhydrobacter aerosaccus* ACCA2 JX042472) fungus (*Aspergillus* sp. ALUH KT356201), earthworm (*Eisenia foetida*) and blackgram (*Vigna mungo*) cv., vamban 4 were obtained from the Department of Agricultural Microbiology, Department of Soils and Environment, Agricultural College and Research Institute, Tamil Nadu Agricultural University, Madurai, Tamil Nadu, India, and National Pulses Research Station, Vamban, Tamil Nadu, India respectively. The bacterial culture (*Enhydrobacter aerosaccus* ACCA2) was grown in Carboxy methyl cellulose (CMC) liquid medium for 24–48 h at 120 rpm in 30 °C until the population reached 10^9^ cfu mL^−1^. Similarly, the fungi culture (*Aspergillus* sp.) was grown in CMC liquid medium for 72 h at 30 °C until the population reached 10^6^ cfu mL^−1^.

#### 2.1.2. Compost of Rice Husk

RRH was milled into smaller size and composting process was carried out in a smaller cement tank with the dimension of 2.5 × 1.5 × 1.0 m. A layer of small stones was spread to a height of 3 cm above, where a layer of soil (red earth) was added to a height of 5 cm. Twelve kilograms of, RRH was filled in a tank and treatment was imposed as detailed below: (T_1_) RRH (Control); (T_2_) RRH + *Enhydrobacter aerosaccus*; (T_3_) RRH + earthworm; (T_4_) RRH + *Enhydrobacter aerosaccus* + earthworm; (T_5_) RRH + *Aspergillus* sp.; (T_6_) RRH + *Aspergillus* sp. + earthworm; (T_7_) RRH + *Enhydrobacter aerosaccus* + *Aspergillus* sp.; (T_8_) RRH + *Enhydrobacter aerosaccus* + *Aspergillus* sp. + earthworm. After mixing all the inputs, watering was done to maintain moisture. *Enhydrobacter aerosaccus* and *Aspergillus* sp. inoculum was added at the rate of 20 mL/kg of RRH (2%). The compost material was stirred periodically once every 15 days to allow more aeration inside the material. The level of moisture was maintained at 60% WHC on weight basis by adding water at different levels of composting. The substrate was allowed to compost for 3 months and analysed periodically. RRH the primary substrate used for the composting process contained 25.95% of organic carbon (OC), 0.31% of total nitrogen (N), 0.19% of phosphorus (P), 0.22 and 0.05% of potassium (K) and sulphur (S), respectively. Initially C:N ratio and C:P ratios were 85:1 and 135:1, respectively. The pH of RRH was neutral (7.1) and non saline with Electrical Conductivity (EC) of 0.25 dS m^−1^. The calcium (Ca) and magnesium (Mg) content were 0.17 and 0.52%, respectively with a moisture content of 7.60%.

#### 2.1.3. Changes in Physico-Chemical Properties during Composting of Rice Husk and Characterisation

Samples were collected periodically from each treatment on 0, 15, 30, 60 and 90 days for physico-chemical analyses. The compost samples were dried and grounded to pass through 2 mm sieve for chemical analysis of organic carbon, nitrogen, phosphorus and potassium. Total organic carbon in the compost was measured by Walkley-Black chromic acid wet oxidation method and total N was measured by Kjeldhal’s digestion method. Total P content was determined by using spectrophotometer. Total potassium content was estimated on flame photometer by direct feeding. To assess the compost maturity, starch iodine test was performed. Briefly, one gram of powdered compost sample was placed in a beaker and a few drops of ethanol were added to wet the samples. Twenty ml of perchloric acid was added to the samples, stirred and filtered through a filter paper. With few drops of the filtrate, 2 drops of iodine reagent was added. Matured compost gives a yellowish colour and very little precipitate; poor or immature compost gives dark colour and heavy precipitation [11]. Compost C:N and C:P ratios and the nutrient contents were used as a selection criteria and based on this the treatment five consisted of RRH + *Aspergillus* sp. is considered as quality compost and used in further experiments. The morphology of RRH and composted rice husk (CRH) was examined in scanning electron microscope (SEM of FEI, Quanta 200, The Netherlands).

### 2.2. Pot Experiment

#### 2.2.1. Experimental Design

To assess the relative impact of CRH on crop growth, a pot culture experiment was conducted in the screen house of Department of Soils and Environment, Agricultural College and Research Institute, Madurai, Tamil Nadu, India (Latitude 9°54′ N, Longitude 78°54′ E) during October to December 2015 with short duration blackgram Vamban 4 as test crop, in a completely randomized block design. The soil samples were collected from the field, where rice crop was harvested during January 2012 and left fallow due to water shortage. Soil sample for pot experiment was collected during September 2015 and no legume crops were previously cultivated in this soil for three years before sample collection. Experiments were conducted with two different soils viz., non-calcareous black soil of (Typic Haplustalf) and non-calcareous red soil of (Typic Rhodustalf). For each soil, 11 treatments were set with 3 replications; each replication consisted of five pots totalling 165 pots per soil type. The treatment consists of different combinations viz., CRH alone (T_1_); 50% RDF (T_2_); 100% RDF (T_3_); CRH + *Rhizobium* (T_4_); CRH + phosphobacterium (T_5_); CRH + *Rhizobium* + phosphobacterium (T_6_); CRH + 50% RDF (T_7_); CRH + 50% RDF + *Rhizobium* (T_8_); CRH + 50% RDF + phosphobacterium (T_9_); CRH + 50% RDF + *Rhizobium* + phosphobacterium (T_10_); absolute control (T_11_). The pots were filled with 10 kg of soil and 2 seeds of test crop were sown in each pot. At the end of the composting period (90 days), compost was collected and applied in a pot @ 5 t ha^−1^ of soil. Lignite based formulation (LBF) of *Rhizobium* BMBS and phosphobacterium PSB-1 (*Bacillus megateirum* var. *phosphaticum*) each contained 10^9^ CFU/gram were obtained from Department of Agricultural Microbiology, Tamil Nadu Agricultural University, Madurai, Tamil Nadu, India.

#### 2.2.2. Blackgram Growth and Nutrient Status Analyses

Soil samples were drawn from each treatment at critical growth stages such as vegetative (15 Day after Showing (DAS)), flowering (45 DAS), pod initiation (65 DAS) for nutrients analyses. The sample was shade dried, powdered gently with a wooden mallet and sieved through a 2 mm sieve. The sieved sample was analysed for pH, EC, available nutrients and micronutrients by following the standard procedures.

The pH of the sample was determined in 1:2 soil water suspensions using a combined pH meter. The EC of the sample was determined in 1:2 soil-water suspensions using a conductivity meter. The organic carbon of the soil sample was determined by the chromic acid wet digestion method. The soil available N content was estimated by alkaline potassium permanganate method [12]. The soil P content was estimated by ascorbic acid method which was measured colorimetrically using a red filter at 660 nm [13]. The available K content in the soil was extracted by using neutral normal NH_4_OAc and the concentration of K ions in the solution was determined using flame photometer (Systronics, Flame photometer 128) [14]. Available micronutrients in the soil viz., Fe, Mn, Zn and Cu were extracted using Diethylene Triamine Penta Acetic (DTPA) acid and subsequent measurement of micronutrient concentrations in the filtrate were done by using an Atomic Absorption Spectrophotometer (Thermo scientific, ICE 3000 series).

Plant samples at every growth stage were collected and the dry weight of the plants was recorded. At sampling time, plants were uprooted from the pot carefully without breakage of roots and were flooded with water to loosen soil from the plant. Root length was measured from the base to the tip of the lengthiest root and expressed in centimeters. The total numbers of nodules during flowering stage were counted numerically. Number of root hairs was recorded from root region and expressed in numbers. One hundred grains of blackgram from each treatment was counted at random and weighed in an electronic balance and expressed in grams (g). Pods collected from the different treatments were dried, threshed and grain yielded at 12% moisture was recorded for each treatment and expressed in grams (g). Haulms are removed completely, and sun dried to attain a constant weight and then oven dried at 70 °C for each treatment and the yield was recorded and expressed in kg ha^−1^. Blackgram seed and haulms N, P, K content were measured using the standard protocol.

### 2.3. Statistical Analysis

Throughout the study, three replications of each treatment were kept. The data collected were statistically analysed by analysis of variance (ANOVA) using the general linear model version developed by SAS institute Inc., Cary, NC, USA (SAS 2004). Means were compared using the least significant difference (LSD) *p* ≤ 0.05.

## 3. Results and Discussion

### 3.1. Composting of Raw Rice Husk

#### 3.1.1. Changes of Physico-Chemical Properties during Composting of Rice Husk

In the present study, an attempt was made to compost the RRH with microorganisms (fungi and bacterium) in different combinations. Among the different treatments, composting RRH with 2% lignocellulolytic fungus (LCF) significantly increased the nutrient content (NPK) and decreased the OC content, followed by vermicomposting with 2% LCF. The decomposition of RRH into compost by fungal inoculum (*Aspergillus* sp.) might be attributed to effective conversion of silica content of RH into nutritionally rich compost thereby leading to economical and environmentally friendly disposal of crop residue [15].

The composting was carried out for a period of three months and periodical physical observations and chemical analyses were made. The preliminary analysis of samples taken after 15 days of RH decomposition is presented in Table 1 showing lower nutrient content and high C:N and C:P ratios. The change in colour from yellow to black was well observed from day 30 onwards in the compost tank which received 2% lignocellulolytic fungal inoculum. The colour of the final matured compost was dark brown and odour was earthy in nature. The dark brown colour and earthy nature is due to the formation of humic substances. The texture of the compost was granular and the presence of the raw material (RH) was not visible in the CRH.

pH is an important parameter controlling the availability of nutrients such as P, Fe, and Zn. It should get due attention while recommending the compost to be mature and safe for soil application. During the composting of raw rice husk (initial pH-7.1) in the earliest stage, the pH value decreased to 6.1 due to production of organic acids derived from the intense fermentation. Afterward, the pH began to rise and reached 8.6 at the thermophilic period. The increase in pH probably resulted from the release of ammonia due to the proteolytic process [15]. During the cooling down and maturation stage, pH dropped to 7.2–7.8 in all the treatments. Rice husk amended fungal inoculated treatment (T5) had pH of 7.2 which was lower compared to its uninoculated counterpart (pH 7.8). Electrical conductivity, a measure of dissolved salts ranged between 0.25 to 0.27 dSm^−1^, and was far below the safe limit of 3 dSm^−1^ and no remarkable change was observed.

The OC content of compost samples showed a decreasing trend. Initially, the carbon content was high and due to the activity of microorganisms and earthworm, the OC was reduced during the final stage and the treatment with 2% lignocellulolytic fungal inoculation recorded the lowest OC content of 16.01%. The reduction in the OC content of the compost indicated the mineralization of organic materials present in the RH [15]. The result of this experiment was in line with Dashtban et al. [16], who reported enhanced carbon mineralization by fungi during the composting of rice straw.

The total N content during the composting process increased over the days measured. Composting with fungal inoculum recorded the maximum nitrogen content. On day 90, 2% lignocellulolytic fungal inoculum recorded the significantly higher nitrogen content (1.10%). The results of the study are in accordance with Goyal and Sindhu [17]. The N content improved due to a concentration effect caused by degradation of labile OC compounds which reduced the weight of the composting mass. It is believed that when organic matter reduction is more than the loss of NH_3_, nitrogen concentration usually increases. Similarly, the dynamics of P and K showed an increasing trend over the period of composting due to mineralization. Previous studies suggested that microorganism processed waste material contains high concentration of exchangeable K due to enhanced microbial and enzymatic activity during the microbial composting process, which consequently enhances the rate of mineralization [18].

A remarkable change in the C:N ratio was noticed during the composting of RRH. The CRH produced with 2% LCF attained a C:N ratio of 14:1 at end of day 90. The improvement in N and lowering of OC resulted in the lowering of the C:N ratio, which is an important criterion for a compost to be fully mature, as well as being an adequate predictor of the impact of amendments on N cycling on its incorporation in soil. The results proved the effectiveness of the added fungal inoculum and its supplementation in degradation of RH. The above findings were concurrent with the findings of Kumar et al. [15], who have reported the lower C:N ratio (14.6:1) in paddy straw amended with microbial consortium. Eiland et al. [19] reported that the improvement in N and lowering of C as is an important criterion to assessing the maturity of compost.

Compost maturity is defined as the status of biological stability of compost conferring immediate improvement of soil productivity. On day 75 of the composting period, all the compost samples were subjected to the starch iodine test. This test was done to terminate the composting process. A yellow coloured solution without any precipitate was observed in the treatment which received 2% LCF which revealed the compost maturity on day 90. Based on the nutrient dynamics and variation in C:N ratio over the period of composting, the RH inoculated with 2% LCF was concluded as the most matured CRH with good stability and maturation. Similarly, the starch iodine test was used to test the maturity of an empty fruit bunch of palm compost [20].

#### 3.1.2. Structural Changes of Rice Husks during Composting Process (SEM)

**Figure 1:**
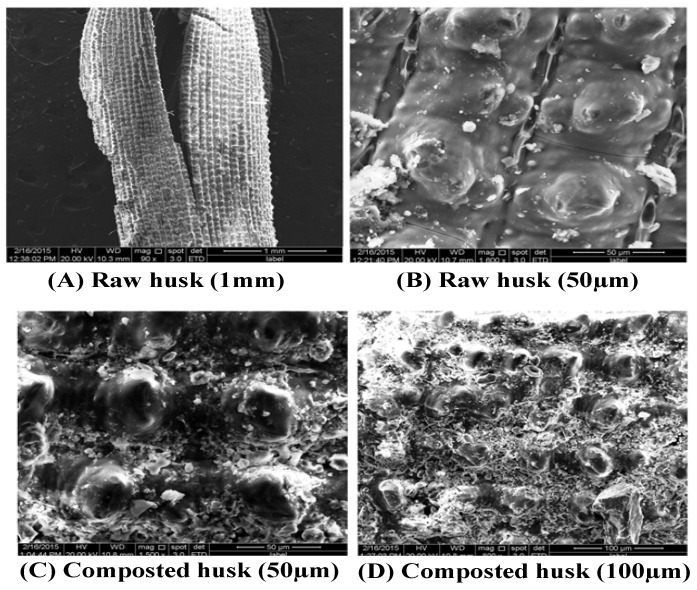
Raw rice husk and the degradation stage of rice husk due to inoculation of lignocellulolytic microorganisms. SEM image of raw rice husk with two different magnifications (**A**,**B**); composted rice husk (**C**,**D**) with two different magnifications.

To determine the changes in the physical structure, a scanning electron microscopic analysis was performed. The micrograph of Figure 1 revealed the surface structure of RRH before and after composting. Moreover, many silica bodies which were found on the surface strand of RRH at the initial stage were removed when the composting was achieved. As clearly shown in Figure 1, the epidermis of RH became loose, rugged and lumpy because the composition of RRH viz., cellulose, hemicellulose, lignin and pectin were decomposed by microbial penetration. The removal of silica bodies would enhance the microbial penetration in the composting process. An earlier study by Baharuddin et al. [21] reported the removal of silica bodies due to the active microbial degradation in oil palm empty fruit bunch degradation. Similarly, SEM was used to access the maturity of flower waste compost [22].

Based on the physico-chemical properties of CRH produced with LCF @ 2% is considered as the best compared to composting of rice husk with earthworm and hence taken for the second phase of the study i.e., to assess the relative efficiency of the CRH on the growth and yield of blackgram in Typic Haplustalf and Typic Rhodustalf.

### 3.2. Effect of CRH on Blackgram Growth Promotion and Soil Properties

#### 3.2.1. Soil Properties

The soil available N status showed a declining trend over the growth stages in both experimental soils (Table S1). The maximum available N of 225 kg ha^−1^ and 280 kg ha^−1^ at harvest stage was recorded in the CRH @ 5 t ha^−1^ with 50% RDF and biofertilizers (T_10_) in Typic Haplustalf and Typic Rhodustalf soils, respectively. In addition, there was a significant increase in available P and K over control in both soils at different growth stages (Table S1). The maximum available phosphorus of 19.92, 18.30 and 17.11 kg ha^−1^ in Typic Haplustalf and 23.21, 21.19 and 18.74 kg ha^−1^ in Typic Rhodustalf was recorded by CRH + 50% RDF + *Rhizobium* + phosphobacterium (T_10_) at vegetative, flowering and at harvest stages, respectively. This might be due to the favourable effect of the combined application of *Rhizobium* and phosphobacterium, which leads to increased availability of nutrients [23]. Available K of 297, 287 and 278 kg/ha in Typic Haplustalf and 307, 297 and 281 kg/ha in Typic Rhodustalf was recorded by during vegetative, flowering and post-harvest stages, respectively. The organic acid formed during the decomposition of RH could have helped in the release of mineral bound insoluble K and it also could have reduced the potassium fixation (Table S1).

#### 3.2.2. Growth Attributes

Application of CRH @ 5 t ha^−1^ with 50% RDF plus biofertilizers @ 2 kg ha^−1^ recorded the maximum root length and root hairs in both experimental soils as reported in Table S2. Significantly higher root length of 12.61, 19.82 and 25.86 cm in Typic Haplustalf and12.87, 22.50 and 26.69 cm in Typic Rhodustalf was recorded in CRH + 50% RDF + *Rhizobium* + phosphobacterium treatment at vegetative, flowering and harvest stages, respectively compared to control. Similarly, CRH + 50% RDF + *Rhizobium* + phosphobacterium treatment recorded the maximum root hairs in critical growth stages of the crop. This might be attributed to the microorganism which secretes certain organic substances, such as auxins, gibberellins, cytokinins, which function as plant growth regulators and influence physiological process resulting in better growth. Similarly, the root nodules of about 26 in Typic Haplustalf and 28 in Typic Rhodustalf numbers were found in CRH + 50% RDF + *Rhizobium* + phosphobacterium treatment during flowering stage compared to control. The combined inoculation of *Rhizobium* plus phosphobacterium plus inorganic fertilizers (50% RDF) with CRH resulted in the significant increase in nodule numbers compared to the single inoculation of *Rhizobium* or PSB with CRH. Similarly, application of *Rhizobium* combined with compost significantly increased nodule number compared to the *Rhizobium* alone treatment [24].

#### 3.2.3. Yield Attributes

The maximum number of branches, pods per branches and grains per pod were noted in treatment CRH + 50% RDF + *Rhizobium* + phosphobacterium (T_10_) compared to control in both the experimental soils (Table 2). Application of biofertilizers increased the plant height and number of branches per plant [25] in pulses. It was observed that there was no significant effect on grain weight in Typic Haplustalf and Typic Rhodustalf. The highest number of branches per plant (2.68 in Typic Haplustalf and 3.78 in Typic Rhodustalf) was noted in CRH + 50% RDF + *Rhizobium* + phosphobacterium (T_10_). This might be attributed to combined effect of CRH with inorganic and biofertilizers application which showed their synergistic effect on crop growth in both Typic Haplustalf and Typic Rhodustalf. Similarly, combinations of *Rhizobium* and Phosphate Solublizing Bacteria (PSB) increased pod number and yield [26].

#### 3.2.4. Grain and Haulm Yield

Application of CRH + 50% RDF + *Rhizobium* and phosbacterium (T_10_) produced the maximum seed yield of 988 and 994 kg ha^−1^, respectively in Typic Haplustalf and Typic Rhodustalf (Table 3). The yield attributes viz., pods per plant and seeds per pod showed an improvement over application of biofertilizers individually. An increase of 51% and 53% grain yield was recorded over control in both soils. This might be due to increased availability of macro and micronutrients in CRH with biofertilizers and inorganic fertilizers which steadily release the nutrient compared to inorganic fertilizers and prevent fixation of nutrients in soil. The similar results of grain yield were reported by Javaid et al. [27] in blackgram and Davari et al. [28] in green gram crop due to different combinations of NPK and organic fertilizers. *Rhizobium* inoculation increased the protein accumulation in the seeds as nitrogen fixation by this bacterium increased the production of protein molecules which contributed to yield properties.

Haulm yield on blackgram was also significant over control in CRH + 50% RDF + *Rhizobium* + phosphobacterium (T_10_). It recorded the maximum haulm yield of 1712 and 1752 kg ha^−1^ in Typic Haplustalf and Typic Rhodustalf, respectively. The combined treatment effect on other yield parameters simultaneously might have contributed to maximum haulm yield. Similarly, the haulm yields of blackgram were markedly increased due to the application of organic manure [29]. An increase of 59% in Typic Haplustalf and 60% in Typic Rhodustalf of haulm yield was recorded by CRH + 50% RDF + *Rhizobium* + phosphobacterium (T_10_) over control.

#### 3.2.5. Protein and Nutrient Content

The current results of this study on crude protein showed the maximum percentage in treatment combination of CRH + 50% RDF + *Rhizobium* and phosphobacterium of 20.19 and 21.44% in Typic Haplustalf and Typic Rhodustalf, respectively, as depicted in Table 3. The increase in protein content is due to the combination of biofertilizers which produced one new protein band [30]. Similar reports of increase in yield attributes, grain yield, total dry matter production and protein content were reported when biofertilizers and organic manures were applied together [31,32].

**Figure 2:**
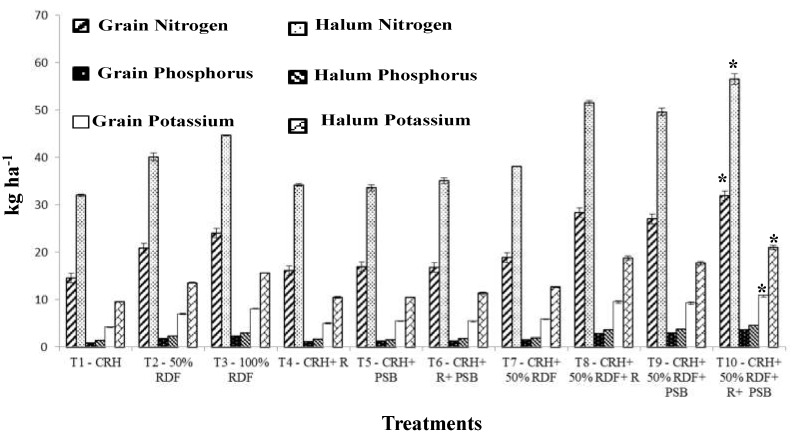
Effect of composted rice husk (CRH) on nitrogen, phosphorus, potassium uptake (kg ha^−1^) by grain and halum of blackgram in Typic Haplustalf. * indicates significant different from other treatment.

**Figure 3:**
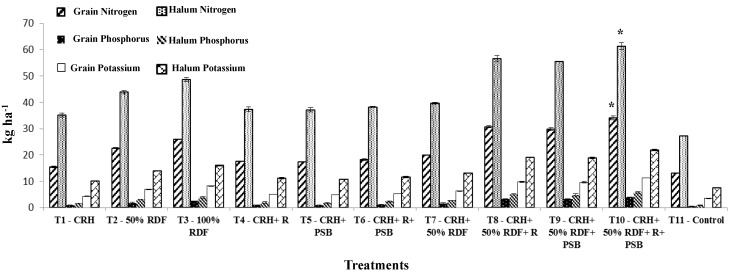
Effect of composted rice husk (CRH) on nitrogen, phosphorus, potassium uptake (kg ha^−1^) by grain and halum of blackgram in Typic Rhodustalf. * indicates significant different from other treatment.

The yield of the crop is generally mediated through the absorption of nutrients which is reflected in the nutrient concentration in different parts of the plant. Irrespective of growth stages and experimental soils used in this study, nitrogen content was found to be higher in all the treatments compared to absolute control. Similarly, the nitrogen content in grain and haulm was also significant over control and registered the maximum N content of 3.23 and 3.30% in Typic Haplustalf and 3.43 and 3.50% in Typic Rhodustalf soil, respectively. Application of CRH + 50% RDF + *Rhizobium* and phosphobacterium each at 2 kg ha^−1^ (T_10_) registered enhanced N content at growth stages compared to other treatments in Typic Haplustalf and Typic Rhodustalf soil (Figure 2 and Figure 3). The increased N content might be attributed to the production of plant nutrients supplied through the integrated use of compost with inorganic and biofertilizers which might have led to the maximum absorption and translocation of N. In this study, *Rhizobium* inoculation increased the nutrient accumulation in seeds by fixing atmospheric nitrogen. Similarly, nitrogen accumulation in soybean seed was significantly increased by *Rhizobium* sp. BARIRGm901 inoculation compared to control [33]. The results of this study were found to be corroborated with the findings of Davari et al. [28], who studied the influence of organic material, crop residues and biofertilizers on performance of the mung bean crop.

Application of CRH + 50% RDF + *Rhizobium* and phosphobacterium (T_10_) each at 2 kg ha^−1^ registered the maximum P content during all the growth stages. Likewise, the maximum P content in grain of 0.37 and 0.40% in Typic Haplustalf soil and Typic Rhodustalf soils, respectively. In haulm, 0.27% P in black soil and 0.33% P in Typic Rhodustalf soil were also marked by (T_10_). The phosphorus content recorded by 50 and 100% RDF were significantly different from CRH + *Rhizobum* + phosphobacterium (T_6_) in both soils. This may be ascribed to the solubilizing effects of P in the soil by organic acids produced during the decomposition of manures and enhanced the release of nutrient into the soil, which in turn might have increased the P content in grain and haulm. This is in close agreement with the findings of Sangeetha et al. [29] in blackgram.

The K content, similar to N and P content, was also significantly increased over absolute control at all growth stages in both the experimental soils. The maximum K content in grain and haulm was registered by soil application of CRH + 50% RDF + *Rhizobium* and phosphobacterium (T_10_) each at 2 kg ha^−1^. This might be due to the continuous supply of nutrients by the enriched compost that leads to better K accumulation in grain and haulm. T_10_ was followed by CRH + 50% RDF + R (T_8_) and CRH + 50% RDF + R (T_9_) which was on par with each other and showed significant difference from 50 and 100% RDF. More effective supply of nutrition was observed with combined application of N fixing bacteria and PSB in blackgram than individual application [34].

## 4. Conclusions

This research is a first attempt to compost RRH with microbial cultures and evaluate its efficiency in improving the soil nutrient status as well as the yield of crop.

It could be concluded from the present study, that the RH composted with 2% (LCF) for 90 days can be a viable alternate organic manure to enhance the yield and quality of blackgram crop.

The combined application of CRH @ 5 t ha^−1^ with 50% RDF and biofertilizers @ 2 kg ha^−1^ can be thought to boost the pulse production.

However, research on the low cost of composting of RH at a large scale and the effectiveness of CRH at field levels is yet to be ascertained.

## Figures and Tables

**Table 1:** Changes in chemical parameters of rice husk during decomposition on day 15 and 90.

Treatments	Organic Carbon		Total Nitrogen		Total Phosphorus		Total Potassium		C:N Ratio		C:P Ratio	
	%								Ratio			
	Day 15	Day 90	Day 15	Day 90	Day 15	Day 90	Day 15	Day 90	Day 15	Day 90	Day 15	Day 90
T_1_	25.80 ± 0.63	22.78 ± 0.15	0.31 ± 0.01 ^e^	0.63 ± 0.01 ^g^	0.20 ± 0.00 ^f^	0.61 ± 0.01 ^f^	0.29 ± 0.00 ^g^	0.78 ± 0.02 ^h^	83:1	36:1	129:1	37:1
T_2_	25.65 ± 0.11	20.76 ± 0.52	0.34 ± 0.00 ^b,c^	0.87 ± 0.01 ^e^	0.26 ± 0.00 ^d^	0.78 ± 0.01 ^d^	0.35 ± 0.01 ^e^	0.99 ± 0.02 ^f^	75:1	24:1	99:1	27:1
T_3_	24.60 ± 0.51	20.16 ± 0.14	0.39 ± 0.00 ^a^	0.92 ± 0.02 ^d^	0.32 ± 0.01 ^b^	0.82 ± 0.00 ^c,d^	0.43 ± 0.00 ^c^	1.09 ± 0.03 ^d^	63:1	22:1	77:1	25:1
T_4_	24.82 ± 0.61	19.86 ± 0.37	0.36 ± 0.01 ^b^	0.95 ± 0.01 ^c^	0.33 ± 0.01 ^b^	0.86 ± 0.02 ^c^	0.46 ± 0.01 ^b^	1.14 ± 0.02 ^c^	69:1	21:1	75:1	23:1
T_5_	25.62 ± 0.01	16.01 ± 0.17	0.34 ± 0.00 ^b,c^	1.10 ± 0.00 ^a^	0.29 ± 0.00 ^c^	0.96 ± 0.00 ^a^	0.44 ± 0.00 ^b,c^	1.24 ± 0.03 ^a^	75:1	14:1	88:1	17:1
T_6_	24.72 ± 0.22	17.76 ± 0.45	0.36 ± 0.01 ^b^	1.03 ± 0.00 ^b^	0.36 ± 0.00 ^a^	0.91 ± 0.01 ^b^	0.49 ± 0.00 ^a^	1.19 ± 0.01 ^b^	69:1	17:1	69:1	20:1
T_7_	25.59 ± 0.36	21.77 ± 0.32	0.32 ± 0.01 ^d,e^	0.69 ± 0.01 ^f^	0.21 ± 0.00 ^e^	0.69 ± 0.00 ^e^	0.32 ± 0.00 ^f^	0.82 ± 0.01 ^g^	80:1	31:1	122:1	32:1
T_8_	24.78 ± 0.61	21.26 ± 0.39	0.33 ± 0.01 ^d,e^	0.70 ± 0.01 ^f^	0.24 ± 0.00 ^d^	0.73 ± 0.00 ^e^	0.38 ± 0.01 ^d^	1.04 ± 0.00 ^e^	75:1	30:1	103:1	29:1
LSD (*p* ≤ 0.05)	0.60	0.47	0.02	0.02	0.02	0.04	0.02	0.04	-	-	-	-

(T_1_) Raw Rice Husk (Control); (T_2_) Raw Rice Husk + *Enhydrobacter aerosaccus* (LBF 2%); (T_3_) Raw Rice Husk + Earthworm (Vermicomposting); (T_4_) Raw Rice Husk + *Enhydrobacter aerosaccus* + Earthworm; (T_5_) Raw Rice Husk + *Aspergillus* sp. (LCF 2%); (T_6_) Raw Rice Husk + *Aspergillus* sp. + Earthworm; (T_7_) Raw Rice Husk + *Enhydrobacter aerosaccus* + *Aspergillus* sp.; (T_8_) Raw Rice Husk + *Enhydrobacter aerosaccus* + *Aspergillus* sp. + Earthworm. Data are presented as mean ±SE (standard error) from three replications, letters “^a^”–“^h^” represent significant differences between the treatments.

**Table 2:** Effect of composted rice husk (CRH) on yield attributes of blackgram.

Treatments	*Typic Haplustalf*				*Typic Rhodustalf*			
	Branches Per Plant	Pods Per Branch	Grains Per Pod	100 Grain Weight (g)	Branches Per Plant	Pods Per Branch	Grains Per Pod	100 Grain Weight (g)
T_1_	1.98 ± 0.05 ^f^	2.11 ± 0.03 ^e^	3.01 ± 0.03 ^e^	3.11 ± 0.02 ^g^	5.44 ± 0.04 ^f^	5.60 ± 0.02 ^i^	4.17 ± 0.01	4.15 ± 0.02
T_2_	2.29 ± 0.01 ^d^	2.45 ± 0.03 ^c^	3.20 ± 0.05 ^d^	3.38 ± 0.06 ^d^	5.81 ± 0.10 ^d^	6.42 ± 0.06 ^e^	4.40 ± 0.04	4.33 ± 0.02
T_3_	2.41 ±0.05 ^c^	2.58 ± 0.04 ^b^	3.33 ± 0.07 ^c^	3.50 ± 0.04 ^c^	5.93 ± 0.09 ^c^	6.57 ± 0.05 ^d^	4.52 ± 0.01	4.52 ± 0.07
T_4_	2.12 ± 0.04 ^e^	2.25 ± 0.02 ^d^	3.02 ± 0.03 ^e^	3.19 ± 0.02 ^f,g^	5.59 ± 0.14 ^e^	6.03 ± 0.08 ^h^	4.20 ± 0.07	4.20 ± 0.05
T_5_	2.11 ± 0.05 ^e^	2.23 ± 0.00 ^d^	3.02 ± 0.06 ^e^	3.22 ± 0.04 ^f,g^	5.56 ± 0.14 ^e^	5.97 ± 0.08 ^h^	4.18 ± 0.09	4.18 ± 0.02
T_6_	2.14 ± 0.03 ^e^	2.29 ± 0.01 ^d^	3.03 ± 0.02 ^e^	3.26 ± 0.06 ^e,f^	5.63 ± 0.12 ^e^	6.16 ± 0.07 ^g^	4.25 ± 0.03	4.25 ± 0.03
T_7_	2.26 ± 0.05 ^d^	2.41 ± 0.02 ^c^	3.17 ± 0.06 ^d^	3.36 ± 0.01 ^d,e^	5.74 ± 0.04 ^d^	6.29 ± 0.02 ^f^	4.38 ± 0.02	4.38 ± 0.07
T_8_	2.56 ± 0.04 ^b^	2.65 ± 0.02 ^b^	3.56 ± 0.09 ^b^	3.71 ± 0.03 ^b^	6.25 ± 0.03 ^b^	6.89 ± 0.02 ^b^	4.64 ± 0.05	4.64 ± 0.09
T_9_	2.53 ± 0.04 ^b^	2.63 ± 0.06 ^b^	3.43 ± 0.08 ^c^	3.68 ± 0.07 ^b^	6.12 ± 0.03 ^b^	6.71 ± 0.01 ^c^	4.55 ± 0.11	4.55 ± 0.11
T_10_	2.68 ± 0.03 ^a^	3.78 ± 0.03 ^a^	3.76 ± 0.04 ^a^	3.94 ± 0.01 ^a^	7.16 ± 0.18 ^a^	7.45 ± 0.10 ^a^	4.70 ± 0.02	4.80 ± 0.05
T_11_	1.85 ± 0.04 ^g^	1.98 ± 0.03 ^f^	2.99 ± 0.07 ^f^	3.03 ± 0.06 ^h^	5.33 ± 0.12 ^g^	5.41 ± 0.07 ^j^	4.15 ± 0.03	4.12 ± 0.08
LSD (*p* ≤ 0.05)	0.10	0.11	0.12	0.11	0.10	0.12	NS	NS

T_1_—CRH; T_2_—50% RDF; T_3_—100% RDF; T_4_—CRH + R; T_5_—CRH + PSB; T_6_—CRH + R + PSB; T_7_—CRH + 50% RDF; T_8_—CRH + 50% RDF + R; T_9_—CRH + 50% RDF + PSB; T_10_—CRH + 50% RDF + R + PSB; T_11_—Control. Recommended dose of fertilizer (RDF) = 25:50:25:20 kg of N:P_2_O_5_:K_2_O:S ha^−1^. *Rhizobium* (R) and Phosphobacterium (PSB) @ 2 kg ha^−1^; composted rice husk (CRH) @ 5 tonnes ha^−1^. Data are presented as mean ±SE (standard error) from five replications, letters “^a^”–“^j^” represent significant differences between the treatments.

**Table 3:** Effect of composted rice husk (CRH) on the yield and protein content of blackgram.

Treatments	*Typic Haplustalf*			*Typic Rhodustalf*		
	Grain Yield Per kg/ha	Haulm Yield Per kg/ha	Protein Content %	Grain Yield Per kg/ ha	Haulm Yield Per kg/ha	Grain Crude Protein Content %
T_1_	585 ± 9.89 ^g^	1265 ± 14.55 ^f^	15.50 ± 0.34	579 ± 8.20 ^g^	1284 ± 20.18 ^g^	16.75 ± 0.20 ^e^
T_2_	779 ± 18.65 ^d^	1475 ± 16.12 ^c^	16.75 ± 0.11	783 ± 19.15 ^d^	1477 ± 11.53 ^d^	18.06 ± 0.07 ^c,d^
T_3_	848 ± 11.92 ^c^	1550 ± 39.53 ^c^	17.69 ± 0.07	849 ± 21.65 ^c^	1555 ± 2.43 ^c^	19.06 ± 0.04 ^c^
T_4_	649 ± 15.87 ^f^	1346 ± 1.40 ^e^	15.56 ± 0.12	655 ± 16.02 ^f^	1353 ± 13.38 ^f^	16.88 ± 0.07 ^e^
T_5_	675 ± 5.27 ^e,f^	1318 ± 4.80 ^e^	15.63 ± 0.19	640 ± 7.99 ^f^	1337 ± 10.44 ^f^	17.00 ± 0.11 ^e^
T_6_	670 ± 15.69 ^e,f^	1366 ± 14.22 ^d,e^	15.69 ± 0.17	666 ± 9.71 ^f^	1370 ± 4.28 ^e,f^	17.06 ± 0.10 ^d,e^
T_7_	711 ± 14.06 ^e^	1409 ± 33.00 ^d^	16.56 ± 0.19	725 ± 8.30 ^e^	1417 ± 16.96 ^e^	17.13 ± 0.11 ^d,e^
T_8_	921 ± 7.19 ^b^	1644 ± 22.25 ^b^	19.25 ± 0.21	937 ± 8.29 ^b^	1692 ± 1.76 ^b^	20.30 ± 0.12 ^b^
T_9_	912 ± 8.07 ^b^	1621 ± 30.37 ^b^	18.50 ± 0.25	932 ± 0.49 ^b^	1688 ± 36.90 ^b^	20.00 ± 0.14 ^b^
T_10_	988 ± 23.65 ^a^	1712 ± 17.82 ^a^	20.19 ± 0.36	994 ± 12.42 ^a^	1752 ± 12.77 ^a^	21.44 ± 0.21 ^a^
T_11_	510 ± 9.02 ^h^	1006 ± 10.99 ^g^	13.63 ± 0.28	522 ± 2.17 ^h^	1056 ± 7.69 ^h^	15.63 ± 0.16 ^f^
LSD (*p* ≤ 0.05)	58.78	66.10	1.15	53.51	56.02	1.04

T_1_—CRH; T_2_—50% RDF; T_3_—100% RDF; T_4_—CRH + R; T_5_—CRH + PSB; T_6_—CRH + R + PSB; T_7_—CRH + 50% RDF; T_8_—CRH + 50% RDF + R; T_9_—CRH + 50% RDF + PSB; T_10_—CRH + 50% RDF + R + PSB; T_11_—Control. Recommended dose of fertilizer (RDF) = 25:50:25:20 kg of N:P_2_O_5_:K_2_O: S ha^−1^ *Rhizobium* (R) and Phosphobacterium (PSB) @ 2 kg ha^−1^; composted rice husk (CRH) @ 5 tonnes ha^−1^. Data are presented as mean ± SE (standard error) from five replications, letters “^a^”–“^h^” represent significant differences between the treatments.

## Supplementary Materials

The following are available online at http://www.mdpi.com/1660-4601/15/2/358/s1, Table S1: Effect of composted rice husk (CRH) on soil available nutrient content on different growth stages of Blackgram, Table S2: Effect of composted rice husk (CRH) on growth attributes on different growth stages of Blackgram.
