# Supplementary material for: Exploration of Rice Husk Compost as an Alternate Organic Manure to Enhance the Productivity of Blackgram in Typic Haplustalf and Typic Rhodustalf
Source: Int J Environ Res Public Health. 2018 Feb 17;15(2):358. doi: 10.3390/ijerph15020358 (PMC5858427; doi:10.3390/ijerph15020358)
Supplement: Supplementary file 1 [file ijerph-15-00358-s001.doc]

Supplementary Material

**Table S1.** Effect of composted rice husk (CRH) on soil available nutrient content on different growth stages of Blackgram.

| **Treatments** | ***Typic Haplustalf*** | | | | | | | | | ***Typic Rhodustalf*** | | | | | | | | |
| --- | --- | --- | --- | --- | --- | --- | --- | --- | --- | --- | --- | --- | --- | --- | --- | --- | --- | --- |
|  | **Available nitrogen (kg ha^-1^)** | | | **Available phosphorus (kg ha^-1^)** | | | **Available potassium (kg ha^-1^)** | | | **Available nitrogen (kg ha^-1^)** | | | **Available phosphorus (kg ha^-1^)** | | | **Available potassium (kg ha^-1^)** | | |
|  | **30 DAS** | **45 DAS** | **Post Harvest** | **30 DAS** | **45 DAS** | **Post Harvest** | **30 DAS** | **45 DAS** | **Harvest** | **30 DAS** | **45 DAS** | **Post Harvest** | **30 DAS** | **45 DAS** | **Post Harvest** | **30 DAS** | **45 DAS** | **Post Harvest** |
| T_1_ | 192±2.82^g^ | 187±0.80^f^ | 181±1.68^e^ | 13.10±0.15^f^ | 10.97±0.13^f^ | 8.74±0.20^f^ | 250±1.95^f^ | 234±2.46^e^ | 226±4.48 | 250±4.29^f^ | 242±2.59^f^ | 220±0.77^f^ | 15.68±0.15^f^ | 13.57±0.35^f^ | 11.12±0.05^f^ | 261±5.96 | 250±4.85 | 229±4.66 |
| T_2_ | 207±3.88^d^ | 195±3.65^d^ | 194±1.92^d^ | 15.83±0.29^d^ | 13.71±0.10^d^ | 12.04±0.01^d^ | 259±6.07^d^ | 252±5.77^d^ | 244±3.56^d^ | 265±6.07^d^ | 257±0.67^d^ | 246±1.41^d^ | 18.69±0.43^d^ | 16.51±0.03^d^ | 14.14±0.24^d^ | 272±6.94 | 262±4.77 | 246±6.27 |
| T_3_ | 215±2.46^c^ | 209±4.13^c^ | 202±0.95^c^ | 17.12±0.05^c^ | 15.03±0.02^c^ | 13.62±0.26^c^ | 270±2.53^c^ | 264±3.02c | 256±6.40^c^ | 274±5.42^c^ | 268±5.58^c^ | 257±1.34^c^ | 20.07±0.03^c^ | 18.07±0.02^c^ | 15.62±0.18^c^ | 288±4.35 | 273±0.43 | 258±6.31 |
| T_4_ | 195±1.52^fg^ | 189±0.10^f^ | 184±0.67^de^ | 13.13±0.07^f^ | 11.08±0.20^f^ | 8.79±0.06^f^ | 253±2.24^ef^ | 238±4.09^e^ | 231±1.92 | 251±3.79^f^ | 245±3.95^ef^ | 231±3.85^e^ | 15.73±0.21^f^ | 13.63±0.31^f^ | 11.18±0.24^f^ | 263±1.51 | 254±4.23 | 231±2.64 |
| T_5_ | 194±2.12^fg^ | 188±0.98^f^ | 183±4.10^de^ | 13.18±0.27^f^ | 10.98±0.15^f^ | 8.83±0.18^f^ | 251±2.74^f^ | 236±4.79^e^ | 228±3.68 | 252±0.66^ef^ | 243±3.79^ef^ | 230±2.63^e^ | 15.79±0.33^f^ | 13.72±0.21^f^ | 11.27±0.06^f^ | 260±2.84 | 251±5.88 | 230±0.24 |
| T_6_ | 200±4.27^ef^ | 192±1.40^ef^ | 187±0.49^de^ | 13.23±0.30^f^ | 12.33±0.30^e^ | 8.91±0.06^f^ | 258±5.37^de^ | 240±0.75^e^ | 232±2.90 | 253±2.63^ef^ | 251±6.40^de^ | 233±4.49^e^ | 15.89±0.13^f^ | 13.79±0.24^ef^ | 11.34±0.26^f^ | 264±6.05 | 256±3.33 | 241±1.63 |
| T_7_ | 205±3.73^de^ | 198±2.27^de^ | 194±2.22^d^ | 14.47±0.15^e^ | 12.39±0.01^e^ | 10.45±0.15^e^ | 261±6.52^d^ | 250±1.82^d^ | 242±0.13 | 258±3.09^e^ | 253±5.27^d^ | 237±1.60^e^ | 17.20±0.31^e^ | 15.03±0.34^e^ | 12.74±0.27^e^ | 271±2.54 | 264±0.69 | 244±0.13 |
| T_8_ | 225±5.62^b^ | 220±5.38^b^ | 216±4.16^b^ | 18.64±0.21^b^ | 16.54±0.40^b^ | 15.40±0.38^b^ | 287±6.72^b^ | 276±6.03^b^ | 267±2.08^b^ | 284±4.43^b^ | 278±6.37^b^ | 271±5.50^b^ | 21.47±0.42^b^ | 19.43±0.30^b^ | 16.98±0.22^b^ | 298±2.17 | 286±3.87 | 270±1.69 |
| T_9_ | 223±2.44^b^ | 219±3.99^b^ | 212±5.08^b^ | 18.51±0.39^b^ | 16.45±0.11^b^ | 15.43±0.22^b^ | 285±0.15^b^ | 274±2.00^b^ | 265±5.52^bc^ | 282±6.02^b^ | 277±4.47^b^ | 268±0.42^b^ | 21.75±0.49^b^ | 19.66±0.34^b^ | 17.21±0.43^b^ | 297±6.96 | 284±2.81 | 269±2.10 |
| T_10_ | 235±4.16^a^ | 231±3.61^a^ | 225±0.70^a^ | 19.92±0.44^a^ | 18.30±0.10^a^ | 17.11±0.19^a^ | 296±1.23^a^ | 287±6.57^a^ | 278±6.66^a^ | 293±1.68^a^ | 289±4.21^a^ | 280±0.73^a^ | 23.21±0.33^a^ | 21.19±0.03^a^ | 18.74±0.02^a^ | 307±6.55 | 297±3.40 | 281±0.44 |
| T_11_ | 185±3.95^h^ | 176±1.74^g^ | 174±1.18^f^ | 11.88±0.13^g^ | 9.67±0.13^g^ | 7.15±0.01^g^ | 242±5.92^g^ | 224±2.68^f^ | 215±5.48 | 242±4.79^g^ | 233±1.46^g^ | 210±0.87^g^ | 14.34±0.35^g^ | 12.16±0.27^g^ | 9.71±0.10^g^ | 254±0.66 | 239±1.87 | 218±3.63 |
| LSD (p ≤ 0.05) | 6.70 | 9.26 | 7.21 | 1.02 | 1.18 | 1.49 | 6.06 | 8.20 | 10.01 | 6.28 | 8.29 | 8.45 | 1.24 | 1.26 | 1.32 | 6.10 | 8.13 | 9.08 |

T_1_ –CRH, T_2_ - 50% RDF, T_3_ - 100% RDF, T_4_ - CRH+ R, T_5_ - CRH+ PSB, T_6_ - CRH+ R+ PSB, T_7_ - CRH+ 50% RDF, T_8_ - CRH+ 50% RDF+ R, T_9_ - CRH+ 50% RDF+ PSB, T_10_ - CRH+ 50% RDF+ R+ PSB, T_11_ - Control. Recommended dose of fertilizer (RDF) = 25:50:25:20 kg of N: P_2_O_5_: K_2_O: S ha^-1^ *Rhizobium* (R) and Phosphobacterium (PSB) @ 2 kg ha^-1^; composted rice husk (CRH) @ 5 tonnes ha^-1^. Data are presented as mean ± SE (standard error) from five replications, letters shows significant differences between treatments (columns) according to Least Significant Difference (LSD) test (P≤0.05).

**Table S2.** Effect of composted rice husk (CRH) on growth attributes on different growth stages of Blackgram.

| **Treatments** | ***Typic***  ***Haplustalf*** | | | | | | | ***Typic Rhodustalf*** | | | | | | |
| --- | --- | --- | --- | --- | --- | --- | --- | --- | --- | --- | --- | --- | --- | --- |
|  | **Root length (cm)** | | | **Root hairs (nos)** | | | **Root nodules (nos)** | **Root length (cm)** | | | **Root hairs (nos)** | | | **Root nodules (nos)** |
|  | **30 DAS** | **45 DAS** | **Harvest** | **30 DAS** | **45 DAS** | **Harvest** | **45 DAS** | **30 DAS** | **45 DAS** | **Harvest** | **30 DAS** | **45 DAS** | **Harvest** | **45 DAS** |
| T_1_ | 6.00±0.15^g^ | 10.56±0.22^d^ | 15.80±0.18^e^ | 6±0.03^f^ | 24±0.38^e^ | 34±0.50f | 8±0.04^g^ | 6.20±0.16^de^ | 11.73±0.19^f^ | 17.20±0.12^d^ | 6±0.06^e^ | 28±0.02^e^ | 49±0.83^f^ | 13±0.26^f^ |
| T_2_ | 8.47±0.12^e^ | 14.28±0.19^b^ | 20.04±0.04^bc^ | 6±0.10^f^ | 28±0.15^cd^ | 39±0.18^e^ | 11±0.10^f^ | 8.50±0.08^c^ | 16.80±0.38^d^ | 20.65±0.40^c^ | 7±0.04^d^ | 33±0.72^cd^ | 60±0.81^d^ | 15±0.16^e^ |
| T_3_ | 9.00±0.20^d^ | 15.63±0.21^b^ | 21.92±0.05^b^ | 8±0.13^d^ | 32±0.27^c^ | 49±0.15^d^ | 13±0.03^f^ | 9.64±0.14^c^ | 18.51±0.32^c^ | 22.45±0.04^b^ | 9±0.17^c^ | 36±0.22^c^ | 64±0.83^c^ | 17±0.13^d^ |
| T_4_ | 6.90±0.16f^g^ | 12.37±0.12^c^ | 16.23±0.01^d^ | 7±0.07^e^ | 27±0.10^d^ | 38±0.65^e^ | 18±0.28^d^ | 7.60±0.08^d^ | 13.45±0.06^e^ | 17.60±0.40^d^ | 7±0.11^d^ | 31±0.50^d^ | 52±0.76^f^ | 20±0.07^cd^ |
| T_5_ | 6.51±0.14^g^ | 12.17±0.08^c^ | 17.34±0.36^d^ | 6±0.08^f^ | 26±0.54^d^ | 37±0.73^e^ | 17±0.23^d^ | 7.21±0.12^d^ | 13.05±0.13^e^ | 17.40±0.17^d^ | 6±0.07^e^ | 30±0.23^d^ | 51±1.22^f^ | 18±0.35^d^ |
| T_6_ | 7.48±0.07^f^ | 12.77±0.05^c^ | 18.54±0.25^c^ | 7±0.02^e^ | 27±0.60^d^ | 36±0.58^ef^ | 18±0.27^d^ | 8.00±0.15^c^ | 14.59±0.01^de^ | 18.68±0.18^d^ | 7±0.15^d^ | 32±0.50^d^ | 56±0.47^e^ | 21±0.19^c^ |
| T_7_ | 8.30±0.21^e^ | 13.05±0.16^c^ | 19.50±0.43^c^ | 8±0.04^d^ | 30±0.09^c^ | 46±0.38^d^ | 16±0.21^e^ | 8.26±0.09^c^ | 16.19±0.22^d^ | 20.46±0.33^c^ | 8±0.14^d^ | 34±0.14^c^ | 59±0.43^d^ | 17±0.25^d^ |
| T_8_ | 11.57±0.22^b^ | 18.63±0.45^a^ | 24.61±0.03^a^ | 12±0.19^b^ | 38±0.30^b^ | 54±1.21^b^ | 24±0.52^b^ | 12.13±0.10^a^ | 21.31±0.13^ab^ | 25.10±0.04^a^ | 12±0.27^b^ | 42±0.98^b^ | 74±1.85^ab^ | 25±0.38^b^ |
| T_9_ | 10.44±0.10^c^ | 18.31±0.30^a^ | 23.72±0.28^a^ | 10±0.22^c^ | 36±0.84^b^ | 51±0.80^c^ | 21±0.33^c^ | 10.29±0.26^b^ | 20.72±0.19^b^ | 24.40±0.33^ab^ | 10±0.06^c^ | 40±0.06^b^ | 68±0.32^b^ | 22±0.09^c^ |
| T_10_ | 12.61±0.21^a^ | 19.82±0.42^a^ | 25.86±0.30^a^ | 14±0.24^a^ | 46±0.77^a^ | 63±1.21^a^ | 26±0.47^a^ | 12.87±0.24^a^ | 22.50±0.37^a^ | 26.69±0.13^a^ | 15±0.25^a^ | 51±0.11^a^ | 78±1.10^a^ | 28±0.03^a^ |
| T_11_ | 5.40±0.01^h^ | 9.10±0.11^d^ | 14.30±0.11^e^ | 4±0.06^g^ | 21±0.49^f^ | 29±0.59^g^ | 7±0.05^g^ | 5.50±0.12^e^ | 9.80±0.09^g^ | 15.60±0.34^e^ | 5±0.04^f^ | 24±0.61^f^ | 46±0.22^g^ | 8±0.04^g^ |
| LSD  (p ≤ 0.05) | 0.52 | 1.17 | 1.13 | 0.62 | 2.35 | 2.60 | 1.15 | 0.57 | 1.12 | 1.31 | 0.61 | 2.02 | 2.12 | 1.44 |

T_1_ –CRH, T_2_ - 50% RDF, T_3_ - 100% RDF, T_4_ - CRH+ R, T_5_ - CRH+ PSB, T_6_ - CRH+ R+ PSB, T_7_ - CRH+ 50% RDF, T_8_ - CRH+ 50% RDF+ R, T_9_ - CRH+ 50% RDF+ PSB, T_10_ - CRH+ 50% RDF+ R+ PSB, T_11_ - Control. Recommended dose of fertilizer (RDF) = 25:50:25:20 kg of N: P_2_O_5_: K_2_O: S ha^-1^. *Rhizobium* (R) and Phosphobacteria (PSB) @ 2 kg ha^-1^; composted rice husk (CRH) @ 5 tonnes ha^-1^. Data are presented as mean ± SE (standard error) from five replications, letters shows significant differences between treatments (columns) according to Least Significant Difference (LSD) test (P≤0.05).
